# Impact of Iron Overload and Hypomagnesemia Combination on Pediatric Allogeneic Hematopoietic Stem Cell Transplantation Outcomes

**DOI:** 10.3390/nu17152462

**Published:** 2025-07-28

**Authors:** Debora Curci, Stefania Braidotti, Gilda Paternuosto, Anna Flamigni, Giulia Schillani, Antonella Longo, Nicole De Vita, Natalia Maximova

**Affiliations:** 1 Advanced Translational Diagnostic Laboratory, Institute for Maternal and Child Health-IRCCS Burlo Garofolo, 34137 Trieste, Italy; debora.curci@burlo.trieste.it; 2 Department of Pediatrics, Institute for Maternal and Child Health-IRCCS Burlo Garofolo, 34137 Trieste, Italy; stefania.braidotti@burlo.trieste.it (S.B.); giulia.schillani@burlo.trieste.it (G.S.); antonella.longo@burlo.trieste.it (A.L.); nicole.devita@burlo.trieste.it (N.D.V.); 3 Pharmacy and Clinical Pharmacology Department, Institute for Maternal and Child Health-IRCCS Burlo Garofolo, 34137 Trieste, Italy; gilda.paternuosto@burlo.trieste.it (G.P.); anna.flamigni@burlo.trieste.it (A.F.)

**Keywords:** allo-HSCT, iron overload, hypomagnesemia, pediatric, transplant-related complications, immune reconstitution delay

## Abstract

**Background/Objectives:** Pediatric allogeneic hematopoietic stem cell transplantation (allo-HSCT) is complicated by iron overload and hypomagnesemia, both contributing to immune dysfunction and post-transplant morbidity. The combined impact of these metabolic disturbances on pediatric allo-HSCT outcomes remains unexplored. This study aims to determine whether hypomagnesemia can serve as a prognostic biomarker for delayed immune reconstitution and explores its interplay with iron overload in predicting post-transplant complications and survival outcomes. **Methods**: A retrospective analysis was conducted on 163 pediatric allo-HSCT recipients. Serum magnesium levels were measured at defined intervals post-transplant, and outcomes were correlated with CD4+ T cell recovery, time to engraftment, incidence of graft-versus-host disease (GVHD), and survival within 12 months. Iron status, including siderosis severity, was evaluated using imaging and laboratory parameters obtained from clinical records. **Results**: Patients who died within 12 months post-transplant exhibited significantly lower magnesium levels. Hypomagnesemia was associated with delayed CD4+ T cell recovery, prolonged engraftment, and an increased risk of acute GVHD. A strong inverse correlation was observed between magnesium levels and the severity of siderosis. Iron overload appeared to exacerbate magnesium deficiency. Additionally, the coexistence of hypomagnesemia and siderosis significantly increased the risk of immune dysfunction and early mortality. No significant association was found with chronic GVHD. **Conclusions**: Hypomagnesemia is a significant, early predictor of poor outcomes in pediatric allo-HSCT, particularly in the context of iron overload, underscoring the need for early intervention, including iron chelation and MRI, to improve outcomes.

## 1. Introduction

Allogeneic hematopoietic stem cell transplantation (allo-HSCT) remains a cornerstone therapy for pediatric patients with high-risk hematologic malignancies, immunodeficiencies, and metabolic disorders [1]. At the same time, advances in conditioning regimens, donor matching, and graft-versus-host disease (GVHD) prophylaxis have improved survival, but post-transplant complications, including acute GVHD (aGVHD), infections, and organ dysfunction, continue to contribute significantly to non-relapse mortality (NRM). Therefore, finding ways to prevent or reduce post-transplant complications and transplant-related mortality represents a significant clinical challenge [2]. These factors, often overlooked in clinical practice, may synergistically amplify oxidative stress, organ damage, and immune dysregulation, creating a high-risk milieu for complications such as GVHD and infections. Iron overload, a frequent consequence of chronic transfusions and chemotherapy-induced hemolysis, is increasingly recognized as a driver of oxidative stress, liver injury, and immunosuppression in allo-HSCT recipients. Excess free iron catalyzes the formation of reactive oxygen species (ROS), exacerbating tissue damage in organs such as the liver (sinusoidal obstruction syndrome), pancreas, and bone marrow. Critically, iron homeostasis is disrupted during GVHD, where donor T cell-mediated injury to the intestinal mucosa and liver impairs hepcidin regulation, a key hormone encoded by the Hepcidin Antimicrobial Peptide (HAMP) gene, leading to uncontrolled ferroportin-mediated iron export and systemic iron accumulation [3,4]. This dysregulation may fuel a vicious cycle of inflammation and tissue injury, worsening GVHD severity and infection risk. Despite growing recognition of the detrimental impact of iron overload on transplant outcomes, the full extent of multiorgan siderosis and its clinical consequences in pediatric allo-HSCT recipients remains incompletely understood.

Recent studies underscore that iron overload, primarily driven by transfusion burden and cytotoxic therapy, is highly prevalent after HSCT and is associated with increased risk of complications such as severe aGVHD, delayed immune reconstitution, and organ dysfunction. However, diagnosing and monitoring iron overload present significant challenges, as traditional markers, such as serum ferritin, lack specificity and sensitivity for tissue iron deposition. To address these limitations, the noninvasive magnetic resonance imaging (MRI)-based technique was adopted, enabling precise iron quantification in the liver, spleen, pancreas, and bones. This approach allows for comprehensive pre- and post-transplant assessment of systemic iron overload, facilitating the early detection of siderosis and the timely initiation of chelation therapy, which has been shown to improve transplant-related outcomes [5,6,7,8,9,10].

Nevertheless, while iron overload from transfusions and chemotherapy is now recognized as a modifiable risk factor for adverse outcomes in pediatric allo-HSCT, there are still considerable gaps in our knowledge, especially with regard to the interplay between iron-induced oxidative stress and other metabolic disturbances, such as magnesium imbalance, which also plays a crucial role in immune regulation and inflammation.

No studies have systematically evaluated the interaction between magnesium status and iron overload in this setting or their combined impact on post-transplant complications, such as survival and GVHD. Simultaneously, magnesium deficiency, common in pediatric allo-HSCT due to chemotherapy, calcineurin inhibitors, and renal losses, may further compromise clinical outcomes, particularly by delaying immune reconstitution and increasing the risk of acute GVHD. Magnesium (Mg^2+^) is a cofactor for enzymes critical to DNA repair, energy metabolism, and immune cell function. Preclinical studies suggest Mg^2+^ modulates T cell activation and neutrophil extracellular trap formation, while clinical data link hypomagnesemia to increased infection rates and impaired engraftment [11,12]. Hypomagnesemia is an almost universal complication among children undergoing HSCT, particularly when immunosuppression therapy is used. Recent pediatric transplant literature reports incidence rates of 80–94% in the early post-transplant period ([13], PMID: 16278584). Emerging evidence in adult allo-HSCT recipients associates hypermagnesemia with a higher incidence of aGVHD [13]. However, the interplay between iron overload, magnesium status, and post-transplant outcomes remains unexplored in pediatric populations, despite children’s heightened susceptibility to iron-induced organ toxicity and electrolyte imbalances. This study aims to bridge the critical knowledge gap by evaluating the relationship between serum magnesium levels, iron overload, and their combined impact on immune recovery, GVHD, and survival in pediatric allo-HSCT. By elucidating these interactions, we aim to identify novel biomarkers and therapeutic targets that can mitigate post-transplant morbidity in this vulnerable population.

## 2. Materials and Methods

### 2.1. Study Design

A single-center, observational, retrospective study was conducted at the Pediatric Bone Marrow Transplant Center of the Institute for Maternal and Child Health—IRCCS Burlo Garofolo, Trieste, Italy, following approval from the local Institutional Review Board (reference No. IRB RC 44/24). The study was conducted by the principles outlined in the Declaration of Helsinki.

The primary endpoint was evaluating overall survival (OS) at 12 months post-transplant, based on serum magnesium levels and siderosis grading. The study also examined the relationship between magnesium levels, siderosis severity, and the incidence of GVHD after transplantation. Finally, the impact of baseline and Day 0 magnesium levels on immune reconstitution (CD4+ T cell counts at 100 days post-transplant and the time required to reach a CD4+ count of 500 cells/mm^3^) was evaluated.

### 2.2. Study Population and Data Collection

From January 2010 to December 2024, a total of 163 patients aged 0 to 17 years with malignant or nonmalignant hematological diseases and solid tumors who underwent allogeneic hematopoietic stem cell transplantation (allo-HSCT) were retrospectively enrolled. Patients were excluded if they were 18 years or older at the time of transplant, received autologous HSCT, underwent non-myeloablative conditioning, or were undergoing a second or subsequent transplant. Patients with a follow-up of more than 1 year and available liver iron concentration (LIC)-MRI at our Institute, both before and within 6 months post-transplant, were included.

All patients were treated according to standard myeloablative protocols, which included chemotherapy and radiation dosing, as previously described [14,15,16]. GVHD prophylaxis was conducted using a calcineurin inhibitor for the matched related donor. For the matched unrelated donor (MUD), a calcineurin inhibitor and mycophenolate mofetil were used, along with the addition of post-transplant cyclophosphamide starting in 2013 for cases involving a haploidentical donor [17,18]. Anti-thymocyte globulin was included in the myeloablative conditioning regimen for MUD, haploidentical donors, and certain hemoglobinopathies with related donors. Demographic and clinical characteristics for each patient were collected. Data on transplant-related outcomes were gathered, which included OS, event-free survival, transplant-related mortality, early and late transplant-related complications, neutrophil and platelet recovery, immunological reconstitution, and the incidence and severity grading of acute and chronic GVHD assessed according to standardized criteria by EBMT-NIH-CIBMTR [19].

The immunological reconstitution was evaluated, including the recovery of neutrophils, the amount of white blood cells (WBC), and the count of CD4+ T cells [20,21].

### 2.3. Assessment and Categorization of Iron Overload

MRI with T2-weighted single spin-echo free-breathing sequences was conducted before and after transplant to assess liver iron concentration [6,8]. We calculated the tissue-to-muscle ratio for every sequence to assess tissue iron content. According to the liver iron MR protocol of Gandon et al. (2004), iron concentration values < 36 µmol/g were classified as normal, values of 36–100 µmol/g were classified as mild iron overload, values of 100–200 µmol/g as moderate iron overload, and iron concentration values > 200 µmol/g were classified as severe iron overload [22]. The liver and iron MR protocol and calculation algorithms are available at https://imagemed.univ-rennes1.fr/en/mrquantif/online_quantif.php (accessed on 13 March 2025).

The images were kept in our archives, unmodified, for future consultations. Pre-transplant MRI was performed on average 18 days before transplant. Post-transplant MRIs were obtained at 1 to 6 months. Patients who underwent HSCT were divided into two groups: those with a pre-transplant LIC of 36–100 µmol/g (no siderosis to mild siderosis) and those with a LIC > 100 µmol/g (moderate-to-severe siderosis).

### 2.4. Assessment and Categorization of Serum Magnesium Levels

Laboratory tests, including serum Mg^2+^ levels, were retrospectively collected from medical records at two time points: at the time of Transplant Unit admission (baseline) and on the day of HSCT (day 0). Magnesium levels between 1.66 and 2.26 mg/dL were considered normal. Hypomagnesemia was defined as a serum magnesium level below 1.66 mg/dL. No values exceeded the normal range.

### 2.5. Statistical Analysis

Statistical analyses were performed using R v4.1.0. Continuous variables were reported as medians and interquartile ranges (IQR); categorical variables were reported as frequencies and percentages. Group comparisons used chi-square or Fisher’s exact test for categorical variables and the Kruskal–Wallis test for continuous variables. Spearman correlation was used to assess the relationship between magnesium levels and immune reconstitution. Multivariate analysis was used to evaluate the independence of significant univariate findings. A *p*-value < 0.05 was considered statistically significant.

## 3. Results

### 3.1. Patients

The study included 163 pediatric patients (107 males and 56 females) who underwent allo-HSCT from 2010 to 2024. Table 1 summarizes the detailed patient demographics and transplant characteristics.

### 3.2. Magnesium Levels and Grade of Siderosis as Prognostic Factors for Survival

**Figure 1:**
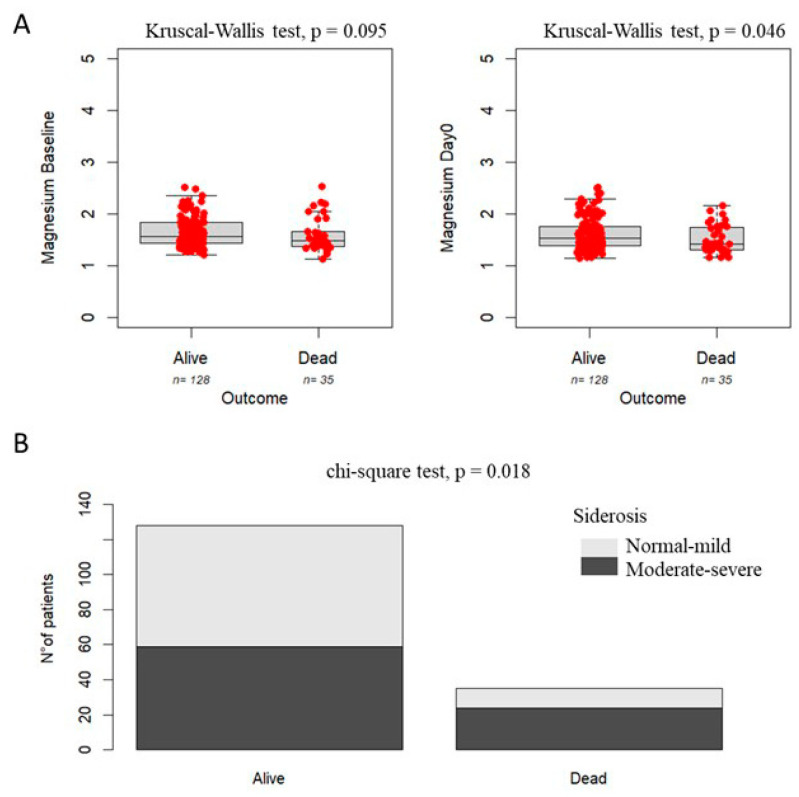
Association between magnesium levels (**Panel A**), grade of siderosis (**Panel B**), and survival in pediatric patients undergoing HSCT.

Figure 1 shows that patients who died within 12 months post-transplant exhibited lower serum magnesium levels compared to survivors, with a trend toward significance at baseline (left panel A: *p* = 0.095) and a statistically significant difference at Day 0 (right panel A: *p* = 0.046). A similar trend was observed when stratifying by the degree of siderosis, with a higher mortality risk (68.6%) in patients with moderate-to-severe siderosis compared to those with mild or absent siderosis (31.4%) (Panel B: *p* = 0.018).

### 3.3. Association Between Magnesium Levels and Siderosis

**Figure 2:**
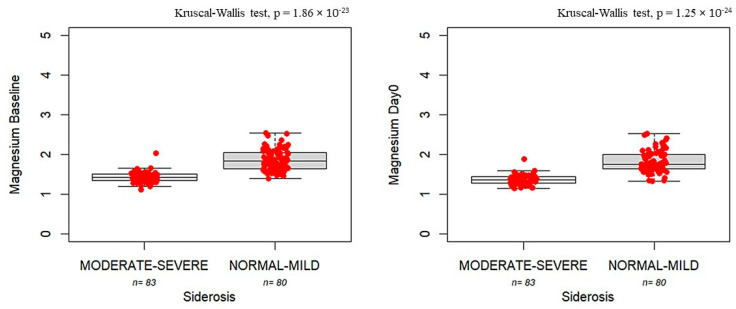
Boxplots showing magnesium concentrations measured at baseline (**left panel**) and Day 0 (**right panel**) in patients with moderate-to-severe siderosis (moderate–severe, *n* = 83) compared to those with normal-to-mild siderosis (normal–mild, *n* = 80). The bold horizontal line represents the median value. Individual red dots represent observed values.

Figure 2 shows that serum magnesium levels at baseline and Day 0 were correlated with the risk of siderosis. Lower magnesium levels at baseline were found for patients with moderate-to-severe siderosis compared with patients with mild siderosis at baseline (1.43 mg/dL, IQR: 0.15 vs. 1.83 mg/dL, IQR: 0.41, *p* = 1.86 × 10^−23^) and at Day 0 (1.36 mg/dL, IQR: 0.16 vs. 1.76 mg/dL, IQR: 0.36, *p* = 1.25 × 10^−24^).

### 3.4. Delayed Immune Reconstitution in Patients with Siderosis and Hypomagnesemia

**Figure 3:**
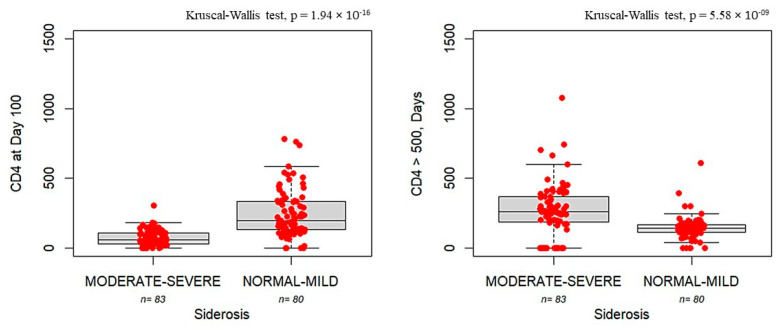
Boxplots showing CD4 cell counts measured on Day +100 (**left panel**) and the number of days required to reach CD4 > 500 cells/mm^3^ (**right panel**) in patients with moderate-to-severe siderosis (moderate–severe, *n* = 83) compared to those with normal iron storage to mild siderosis (normal–mild, *n* = 80). The bold horizontal line represents the median value. Individual red dots represent observed values.

Figure 3 shows that patients with siderosis exhibit delayed immune reconstitution, as evidenced by both reduced CD4+ counts and prolonged time to engraftment compared with patients in normal-to-mild siderosis (CD4 count: 58 [IQR:76] vs. 198 [IQR:205], *p* = 1.94 × 10^−16^; time to engraftment: 258 [IQR:184] vs. 142 [IQR: 52.2], *p* = 5.58 × 10^−9^).

**Figure 4:**
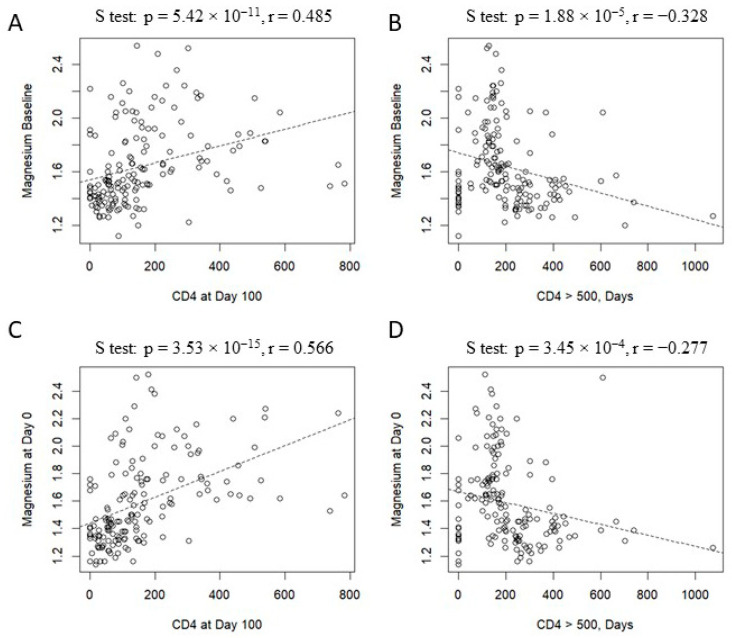
Correlation between magnesium level and immune recostitution: magnesium at baseline vs. CD4 count on Day +100 (**panel A**), magnesium at baseline vs. days to reach CD4 > 500 cells/mm^3^ (**panel B**); magnesium on Day 0 vs. CD4 count on Day +100 (**panel C**); magnesium on Day 0 vs. days to reach CD4 > 500 cells/mm^3^ (**panel D**).

Figure 4 shows delayed immune reconstitution in patients with hypomagnesemia at baseline and Day 0. In particular, a positive association was found between baseline magnesium levels and the number of CD4+ cells (*p* = 5.42 × 10^−11^, r = 0.485, Panel A). In contrast, an inverse trend was observed, considering the day necessary for the achievement of a CD4+ T cell count ≥ 500 cells/mm^3^ (*p* = 1.88 × 10^−5^, r = −0.328, Panel B). The same associations were found, considering magnesium levels on Day 0, the number of CD4+ cells (*p* = 3.53 × 10^−15^, r = 0.566, Panel C), and achieving a CD4+ T cell count ≥ 500 cells/mm^3^ (*p* = 3.45 × 10^−4^, r = −0.277, Panel D).

**Figure 5:**
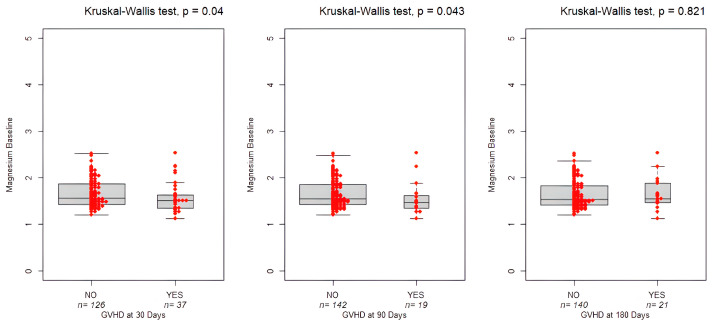
Boxplots showing magnesium levels at baseline and the occurrence of GVHD on Day +30, +90, and +180. The bold horizontal line represents the median value. Individual red dots represent observed values.

Interestingly, as shown in Figure 5, low magnesium levels can lead to an increased risk of aGVHD both on Day 30 and Day 90 (1.5 mg/dL, IQR: 0.43 vs. 1.6 mg/dL, IQR: 0.29, *p* = 0.04 and 1.47 mg/dL, IQR:0.29 vs. 1.55 mg/dL, IQR:0.41, *p* = 0.043, respectively). No association was found between chronic GVHD and the study variables (*p* = 0.821).

## 4. Discussion

This study demonstrated a critical association between serum magnesium levels and early post-transplant mortality, revealing that patients who died within 12 months post-transplant had significantly lower magnesium levels. These findings suggest that hypomagnesemia may serve as an early prognostic biomarker for delayed immune reconstitution, reduced CD4+ T cell counts, and prolonged engraftment in pediatric allo-HSCT recipients, and, with significant clinical implications, provide novel evidence of a potential interplay between magnesium deficiency, iron overload, and impaired immune recovery post-transplant [13].

Hypomagnesemia is increasingly recognized as a critical factor influencing immune function, particularly in HSCT [23,24]. Hematopoietic tissue, which undergoes rapid cellular turnover, relies on magnesium for processes such as DNA and RNA synthesis, as well as cell replication. However, the consequences of intra- and extracellular Mg^2+^ imbalances on hematopoietic tissue remain underexplored [25]. The observed association between low magnesium levels and increased mortality can be explained through several pathophysiological mechanisms [26]. Magnesium is essential for T-lymphocyte activation and natural killer (NK) cell function, and, as reported in our study, can contribute to delayed CD4+ T cell recovery, a critical component of immune surveillance post-transplantation. Additionally, magnesium deficiency can compromise cellular membrane stability and mitochondrial function, potentially increasing susceptibility to infectious complications and GVHD [27]. The role of magnesium in modulating inflammatory responses is particularly relevant in the context of transplantation, where its deficiency may contribute to a chronic pro-inflammatory state that adversely affects outcomes. This finding aligns with several studies examining the role of magnesium in immune reconstitution [28]. Post-HSCT hypomagnesemia often arises from metabolic disturbances and calcineurin inhibitor-induced renal magnesium wasting [29]. Notably, tacrolimus is associated with more pronounced renal magnesium loss than cyclosporine [30,31]. These findings highlight the importance of monitoring magnesium levels and the potential role of supplementation in facilitating immune recovery. A potential pharmacological mechanism contributing to hypomagnesemia in our cohort involves the nephrotoxic effects of calcineurin inhibitors, particularly tacrolimus and cyclosporine, which are commonly used for GVHD prophylaxis. These agents induce renal magnesium wasting primarily by impairing the function of specific channels in the distal convoluted tubule, thereby reducing tubular magnesium reabsorption. Moreover, calcineurin inhibitors can cause subclinical tubular injury, further exacerbating electrolyte disturbances. The strong inverse correlation observed in our study between serum magnesium levels and the severity of siderosis represents a novel finding with important clinical implications. Patients with moderate-to-severe siderosis exhibited significantly lower magnesium levels. Siderosis, indicative of iron overload, also emerged as a key risk factor for immune dysfunction following HSCT.

Several mechanisms may explain this association. Iron overload can impair magnesium absorption in the gastrointestinal tract through competitive inhibition, and it can also increase renal magnesium wasting [32]. Additionally, iron-induced oxidative stress may alter cellular magnesium homeostasis. The use of iron chelators, while beneficial for reducing iron burden, may also contribute to magnesium depletion through chelation or increased urinary losses. Iron regulates macrophage and T cell activity. However, excess iron can drive oxidative stress and inflammation, impairing immune recovery and increasing the risk of aGVHD [4]. Conversely, hypomagnesemia affects iron metabolism, driving iron deposition in organs such as the liver, spleen, heart, and muscles [33,34]. In magnesium-deficient rats, excess dietary iron significantly elevated the saturation of the iron binding capacity, suggesting that magnesium deficiency impairs the homeostatic control of iron transport. In addition, magnesium-deficient rats had low iron levels in the tibia, indicating a decrease in iron in the bone marrow and consequent anemia. These alterations in iron metabolism may suggest that magnesium deficiency leads to inefficient iron utilization, and that iron overload, combined with magnesium deprivation, deteriorates rather than improves iron utilization. Furthermore, unresponsive hepcidin expression may play a role in magnesium deficiency-induced changes associated with increased liver iron [35]. The bidirectional relationship between magnesium deficiency and iron overload creates a potentially harmful cycle where each condition may exacerbate the other, ultimately compromising transplant outcomes. This finding suggests that magnesium supplementation should be considered as part of the comprehensive management strategy for patients with iron overload undergoing allo-HSCT.

Our data confirm that moderate-to-severe siderosis correlates with delayed immune reconstitution, suggesting a synergistic effect between iron and magnesium in driving immune dysregulation. The coexistence of hypomagnesemia and siderosis was linked to a higher risk of GVHD, likely due to combined immune dysregulation. This association suggests that magnesium’s protective effect may be most relevant during the early post-transplant period when acute inflammatory responses predominate. Several mechanisms may underlie this protective effect. Magnesium exerts immunomodulatory activity by suppressing excessive T cell activation and modulating the balance between effector and regulatory T cells, potentially reducing the risk of acute GVHD [36]. Its anti-inflammatory properties—evidenced by the association between magnesium deficiency and increased production of pro-inflammatory cytokines—further contribute to this effect [37]. Additionally, magnesium plays a role in preserving endothelial integrity, and its deficiency may promote the vascular injury characteristic of acute GVHD.

The absence of association with chronic GVHD may reflect the different pathophysiological mechanisms underlying chronic GVHD, which involves fibrotic processes and autoimmune-like phenomena that may be less directly influenced by magnesium status. We acknowledge several limitations of our study. First, its retrospective and single-center design may introduce selection bias and limit our ability to draw definitive causal conclusions. Second, the variability in the timing of MRI assessments and the lack of detailed data on iron chelation therapy may have influenced the evaluation of iron overload and its potential associations. Future prospective studies with larger cohorts and longer follow-up are needed to confirm and expand upon our findings.

## 5. Conclusions

In conclusion, our study highlights the critical roles of hypomagnesemia and siderosis in delaying immune reconstitution and increasing the risk of acute GVHD in pediatric HSCT patients. These results underscore the importance of rigorous monitoring of magnesium levels and iron status after HSCT, as well as the evaluation of therapeutic interventions, such as magnesium supplementation and iron chelation, to enhance immune recovery and reduce GVHD incidence.

Additionally, studies investigating the molecular pathways linking magnesium deficiency, iron overload, and immune dysfunction may identify novel therapeutic targets for correcting these metabolic imbalances and improving transplant outcomes in pediatric HSCT recipients. Addressing these factors through timely clinical interventions may improve post-transplant outcomes and enhance long-term survival in this vulnerable population.

## Figures and Tables

**Table 1:** Demographics and clinical characteristics of allo-HSCT recipients.

Demographics and Clinical Data	Cohort (*n* = 163)
**Age at transplant:**	
Median [IQR], years	8.5 [4–17]
**Gender, number (%):**	
Male, *n*° (%)	107 (66%)
Female, *n*° (%)	56 (34%)
**Primary disease, number (%):**	
Acute lmphoblastic leukemia (ALL)	70 (42.9%)
Acute myeloid leukemia (AML)/Myelodysplastic syndrome (MDS)	31 (19.0%)
Inborn errors	34 (20.8%)
Hemoglobinopathies	26 (15.9%)
Lymphoma	2 (1.2%)
**Allogenic donor type, number (%):**	
Matched unrelated donor	76 (46.6%)
Matched related donor/Sibling donor	68 (41.7%)
Haploidentical donor	19 (11.6%)
**Stem cell source, number (%):**	
Bone marrow	82 (50.3%)
Peripheral blood stem cell (PBSC)	81 (49.7%)
**Myeloablative conditioning regimen, number (%):**	
MCHT-based	111 (68.1%)
TBI-based	52 (31.9%)
**Immunosuppression, number (%):**	
Cyclosporin A	10 (6.1%)
Tacrolimus	148 (90.8%)
Other	5 (3.1%)

MCHT, myeloablative chemotherapy; TBI, total body irradiation; DRIQR, interquartile range.

## Data Availability

The datasets used and analyzed during the current study are available from the corresponding author on reasonable request.
